# Trends in sample preparation and separation methods for the analysis of very polar and ionic compounds in environmental water and biota samples

**DOI:** 10.1007/s00216-020-02811-5

**Published:** 2020-07-24

**Authors:** Sarah Knoll, Tobias Rösch, Carolin Huhn

**Affiliations:** Institute of Physical and Theoretical Chemistry, Eberhard Karls Universität Tübingen, Auf der Morgenstelle 18, Tübingen, Germany

**Keywords:** Hydrophilic interaction chromatography, Capillary electrophoresis-mass spectrometry, Mixed-mode stationary phases, Electromembrane extraction, Supercritical fluid chromatography, Ion chromatography-mass spectrometry, Green analytical chemistry

## Abstract

Recent years showed a boost in knowledge about the presence and fate of micropollutants in the environment. Instrumental and methodological developments mainly in liquid chromatography coupled to mass spectrometry hold a large share in this success story. These techniques soon complemented gas chromatography and enabled the analysis of more polar compounds including pesticides but also household chemicals, food additives, and pharmaceuticals often present as traces in surface waters. In parallel, sample preparation techniques evolved to extract and enrich these compounds from biota and water samples. This review article looks at very polar and ionic compounds using the criterion log *P* ≤ 1. Considering about 240 compounds, we show that (simulated) log D values are often even lower than the corresponding log *P* values due to ionization of the compounds at our reference pH of 7.4. High polarity and charge are still challenging characteristics in the analysis of micropollutants and these compounds are hardly covered in current monitoring strategies of water samples. The situation is even more challenging in biota analysis given the large number of matrix constituents with similar properties. Currently, a large number of sample preparation and separation approaches are developed to meet the challenges of the analysis of very polar and ionic compounds. In addition to reviewing them, we discuss some trends: for sample preparation, preconcentration and purification efforts by SPE will continue, possibly using upcoming mixed-mode stationary phases and mixed beds in order to increase comprehensiveness in monitoring applications. For biota analysis, miniaturization and parallelization are aspects of future research. For ionic or ionizable compounds, we see electromembrane extraction as a method of choice with a high potential to increase throughput by automation. For separation, predominantly coupled to mass spectrometry, hydrophilic interaction liquid chromatography applications will increase as the polarity range ideally complements reversed phase liquid chromatography, and instrumentation and expertise are available in most laboratories. Two-dimensional applications have not yet reached maturity in liquid-phase separations to be applied in higher throughput. Possibly, the development and commercial availability of mixed-mode stationary phases make 2D applications obsolete in semi-targeted applications. An interesting alternative will enter routine analysis soon: supercritical fluid chromatography demonstrated an impressive analyte coverage but also the possibility to tailor selectivity for targeted approaches. For ionic and ionizable micropollutants, ion chromatography and capillary electrophoresis are amenable but may be used only for specialized applications such as the analysis of halogenated acids when aspects like desalting and preconcentration are solved and the key advantages are fully elaborated by further research.

**Electronic supplementary material:**

The online version of this article (10.1007/s00216-020-02811-5) contains supplementary material, which is available to authorized users.

**Figure:**
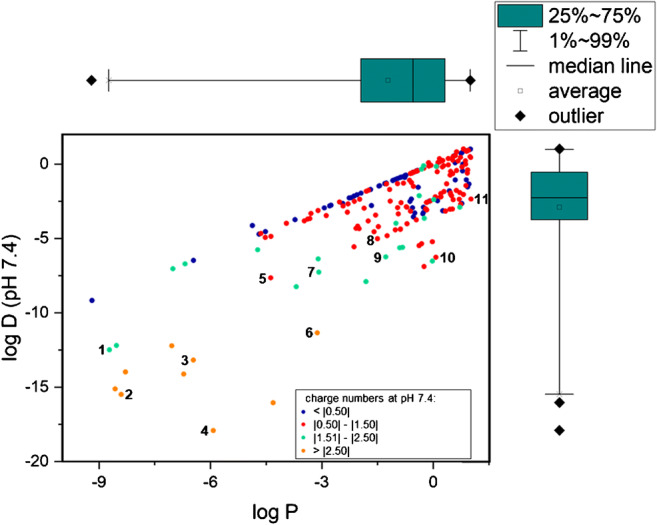
Graphical abstract

## Introduction

**Fig. 1:**
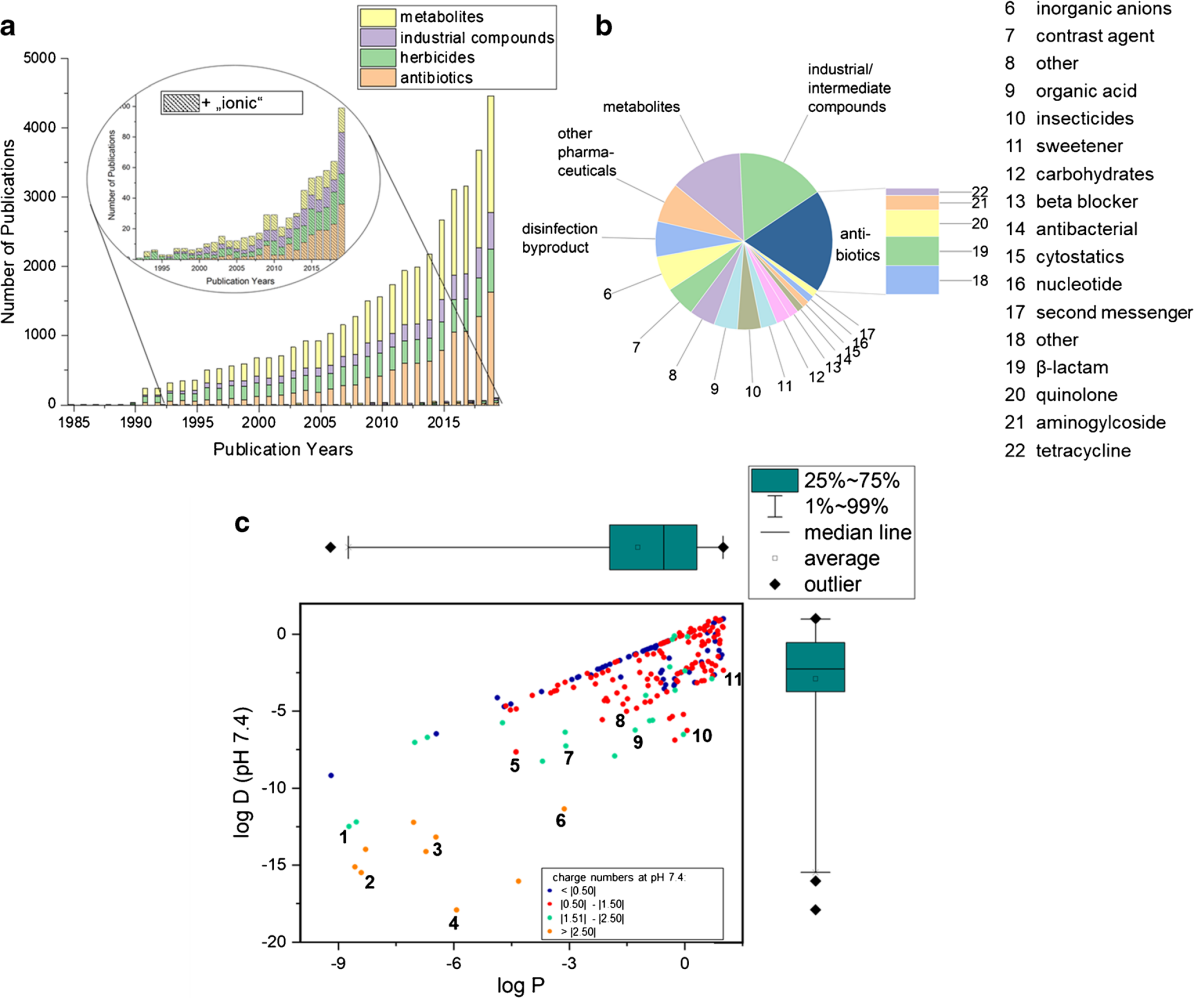
**a** Non-exhaustive trend of numbers of published articles over the last 35 years listed in “Web of Science” using the keywords environmental + most common substance classes (metabolites, industrial compounds, herbicides, or antibiotics, see also **b**) and additionally to it “ionic” in a second search (see enlarged section). **b** Classification of the 237 very polar and ionic compounds (vPICs, log P ≤ 1) analyzed in 63 cited articles. Details of the compounds can be found in Table S1 in the ESM. Only substance classes with ≥ 3 members are included. **c** Scatterplot with marginal boxplot of log P and the corresponding log D (pH 7.4) values of the compounds. Log P and log D data were extracted from Chemicalize provided by ChemAxon (10/05/2020). Colors code for different charge numbers (given as absolute number (modulus)). Selected analytes with large differences between log P and log D (see also ESM Table S1) are (1) Gd-BT-DO3A, (2) neomycin, (3) tobramycin, (4) Gd-DTPA, (5) cephalosporin C, (6) gentamicin, (7) glyphosate, (8) cephalozin, (9) 3-methylphosphinic acid, (10) itaconate, and (11) 3,4-dihydroxyphenylacetic acid

Already in 1985, Richardson and Bowron [1] discussed the fate of pharmaceuticals and other micropollutants in the (aquatic) environment. Over the last 35 years, the interest in their fate steadily increased as can be seen by a statistic for the keywords environmental and most common substance classes (antibiotics, metabolites, but also industrial compounds, herbicides, see Fig. 1a). Recent years showed improvements throughout the whole analytical process. However, due to the different physicochemical properties of micropollutants caused by a variety of functional groups, it is obvious that there is no analytical method for the successful simultaneous analysis of all contaminants in a sample. Whereas for the analysis of non-polar to medium polar compounds (log *P* > 1) successful and reliable analysis methods already exist, there is a lack of methods for the analysis of very polar and ionic compounds (vPICs). Reemtsma et al. [2] even pointed out that—at present—an analytical gap exists for these substances. Despite great effort in the past years in environmental analysis, visible from the rising number of publications (see Fig. 1a, enlarged section), still little is known about the occurrence, fate, and potential ecological effects of vPICs and their mostly even more polar metabolites in the (aquatic) environment. Since these compounds are highly polar, often mobile and possibly also persistent, they have the potential to spread through the water cycle and even reach drinking water. For ionic compounds, the charge influences sorption and uptake, which is not well understood. With increasing awareness of their possible ecotoxicological relevance, e.g., effects on aquatic organisms and health threats, the demand of general public for tightened regulation is rising. This requires further research to increase knowledge and understanding of occurrence and effects. Consequently, sensitive and precise methods for the analysis of these mostly small organic compounds are needed.

We compiled data from recent articles dealing with the analysis of vPICs (log *P* ≤ 1) in the environment. Figure 1 b classifies 237 different vPICs that were analyzed in 63 published cited here with regard to their application. Clearly, vPICs are present in all fields of applications. It is important to mention that this group also contains other micropollutants than pharmaceuticals and their metabolites (which themselves are mostly more polar, e.g., abacavir and its metabolite abacavir carboxylate with log D (pH 7.4) values of 0.38 and − 2.72, respectively (see also Table S1 in the Electronic Supplementary Material, ESM). In fact, only less than about 40% of the compounds analyzed here are pharmaceuticals. Other substance classes like food supplements (e.g., (artificial) sweetener) or industrial compounds in general are quite common representatives for vPICs. In order to get a feeling for the polarity of the substances, Fig. 1c shows the distribution of their log *P* values. Having in mind that at pH 7.4 over 70% of these compounds have charge numbers equal to or higher than 0.5, it is crucial to consider their corresponding log D values at pH 7.4 which are also summarized in Fig. 1c (details are presented in Table S1 in the ESM). A Welch’s *t* test showed a statistically significant difference between the log *P* values and their corresponding log D (pH 7.4) values. Details on the correlation of log P and log D (pH 7.4) are also presented in Fig. 1 c. As expected, the average polarity of these compounds increases (log D decreases) which will result in different behavior in the environment. Several compounds such as gadolinium-based contrast agents or aminoglycoside antibiotics have large differences between log P and log D (pH 7.4) (see Fig. 1c).

**Fig. 2:**
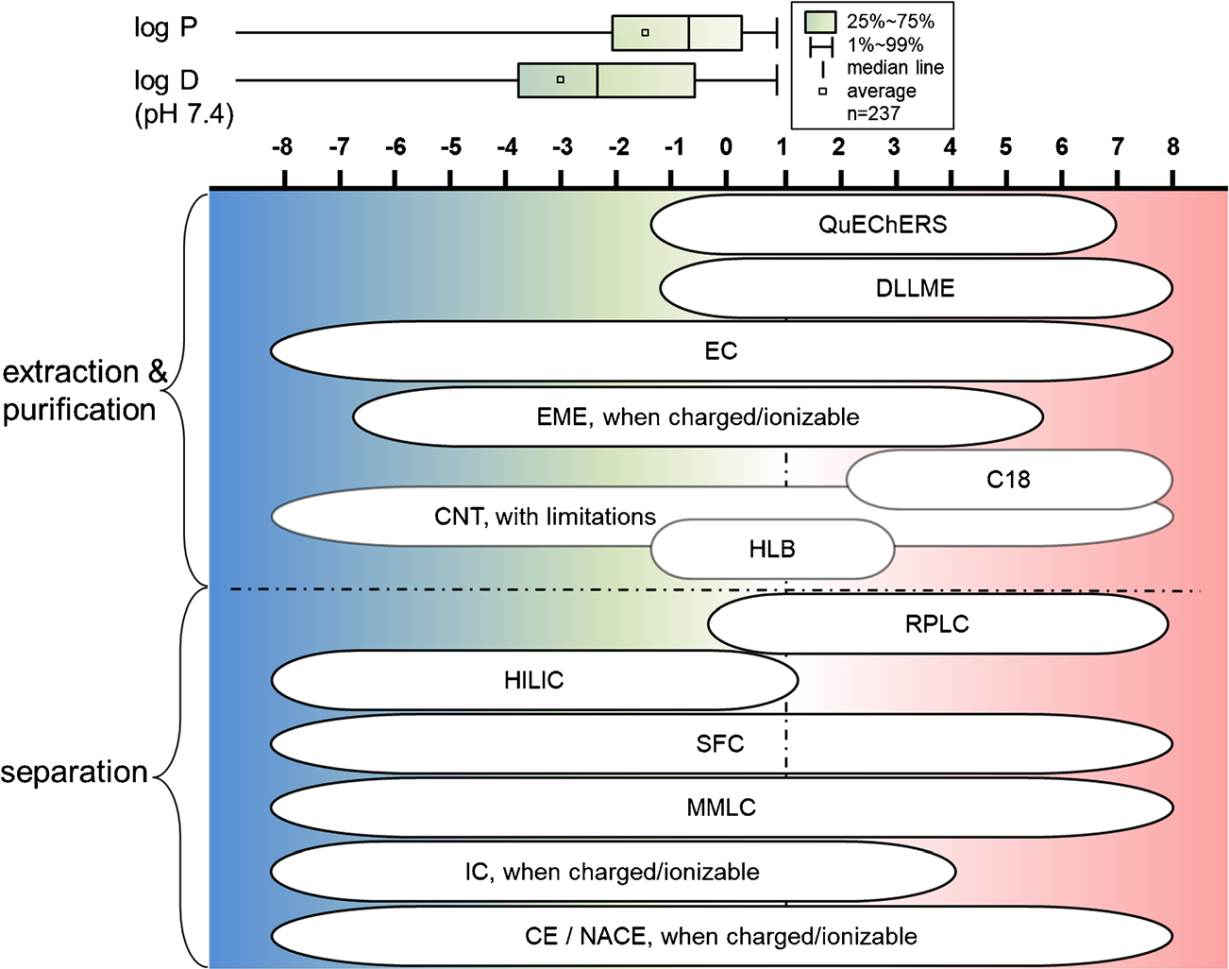
Distribution of log P and log D (pH 7.4) values of the compounds listed in ESM Table S1 (classification according to application as in Fig. 1b). Details of the correlation between log P and log D (7.4) is shown in Fig. 1c). Contours show estimated log P scopes of the sample preparation and separation techniques as discussed in this article. DLLME, dispersive liquid-liquid microextraction; EC, evaporative concentration; EME, electromembrane concentration; CNT, carbon nanotubes; HLB, hydrophilic-lipophilic balance; RPLC, reversed phase liquid chromatography; HILIC, hydrophilic interaction liquid chromatography; SFC, supercritical fluid chromatography; MMLC, mixed-mode liquid chromatography; IC, ion chromatography; (NA)CE, (nonaqueous) capillary electrophoresis

Mass spectrometry (MS) is the detection method of choice to reach the required detection limits, as it provides identification strength and an additional separation dimension when coupled to the various separation techniques [3]. Current screening and environmental monitoring strategies for micropollutants often rely on solid-phase extraction (SPE) for sample preparation and liquid chromatography (LC), mostly C18 reversed phase (RP) stationary phases, for separation. Both SPE and reversed phase liquid chromatography (RPLC) have limitations for vPICs. This is due to insufficient retention, leading to poor extraction efficiencies and poor separation of early eluting peaks in RPLC. Figure 2 provides an overview on current analytical methods (sample preparation and separation) applicable for vPIC analysis and the range of log P/log D values, which can be covered (in part, however, of course not the whole range with a single analysis). Limitations regarding the polarity range covered are evident in many up-to-date reviews [4–7]. Various analytical approaches were tested to address polar micropollutants with hydrophilic interaction liquid chromatography (HILIC) being the most prominent method, followed by supercritical fluid chromatography (SFC), mixed-mode liquid chromatography (MMLC), and combinations thereof. We will discuss the applicability of ion chromatography (IC) and capillary electrophoresis (CE) suited for ionic and ionizable compounds (as the majority of polar compounds is ionizable). New instrumental developments, e.g., for IC-MS, will be discussed and also innovations, e.g., improved stationary phases with a column material particle size less than 2 μm for ultra-high performance SFC (UHPSFC).

Environmental samples, especially biota samples, have complex matrices. Matrix effects seem to be relevant especially for very polar analytes [8]. They have to be addressed enhancing selectivity (both for separation and detection) and using sample preparation strategies. While common sample preparation techniques like SPE are still advancing quickly, multiresidue methods such as QuEChERS (quick, easy, cheap, effective, rugged, and safe) found their way from food to environmental soil and biota analysis. We here want to discuss some alternatives, too, such as electromembrane extraction (EME), some of them only applicable for ionic and ionizable analytes.

In the past, there have been some reviews or trend articles already addressing the analysis of polar compounds [3–6, 9]. In this review article, we will focus on liquid-phase separations coupled to MS and sample preparation strategies for the analysis of vPICs (log *P* ≤ 1) in environmental samples including water and biota. We critically discuss new developments in the field differentiating between targeted and non-targeted methods. We here want to discuss sample preparation in combination with different separation methods, e.g., IC, CE, and SFC, and to elaborate recent trends and emphasize their potential for the analysis of vPICs in environmental samples in the future.

## Trends in sample preparation

### Sample preparation for environmental matrices

Beside robustness and reproducibility, the main objectives of sample preparation are the removal of matrix compounds, analyte recovery, and especially analyte preconcentration, whereas the latter two are also often combined in the term enrichment factor. Since most micropollutants in environmental samples are present at trace levels (ng/l to μg/l range), separation techniques alone are not able to reach these concentration levels, and they can be utilized only if sample enrichment is applied [10–13]. Moreover, smaller initial sample sizes (especially important in biota analysis of invertebrates), improvement in extraction selectivity for targeted analysis, and also coverage in non-target analysis are essential [3]. Coupling to analytical separation methods plays an important role [14]. Another aspect is green analytical chemistry, which has emerged in the 1990s, due to many analytical methods themselves generating a significant amount of chemical waste, resulting in a great environmental impact. It is required to reduce the amount and toxicity of applied solvents and reagents, especially by automation and miniaturization [15]. In this section, we focus on modern approaches for the sample preparation of polar and ionic analytes in water and biota samples.

### Solid-phase extraction

Conventional offline SPE is the method of choice for isolating and enriching organic micropollutants from environmental samples with a wide range of sorbents available [16]. Based on the nature of the analytes, a careful choice of the sorbent material allows obtaining high recoveries and enrichment factors, typically in the range of 2–1000. Of all sorbents available for SPE, Oasis HLB is the most common sorbent used for environmental samples. It is a copolymeric sorbent with hydrophilic and lipophilic properties and can be used for a wide range of target compounds. It is water-wettable and stable over the whole pH range. In environmental analysis, it was used for the extraction of pharmaceuticals of various therapeutic classes from different matrices including wastewater [17, 18], sludge [19], and fish tissue [20]. However, the study of Boulard et al. [21] showed limitations of this material for polar pharmaceuticals and their transformation products with a log D (pH 7) close to or below 0. Alternatives to Oasis HLB are carbonaceous SPE sorbents, as they combine polar and non-polar interactions, making them suitable for a large polarity range (see Zahn et al. [3] for further discussion). In environmental analysis, they were applied for the analysis of polar pesticides [22] and herbicides [23].

Mixed-mode SPE sorbents became commercially available, which are able to retain compounds through both hydrophobic interactions and ionic interactions so that ionic or ionizable compounds can also be extracted [6]. For example, Scheurer et al. [24] used Strata X-CW columns to extract the ionic antidiabetic metformin from sewage and surface water with a relative recovery ≥ 90%. However, even SPE materials intended for polar compounds are often unable to retain the most polar compounds and ion exchange materials are limited to charged compounds. Accordingly, either combinations of different SPE sorbents or alternative methods such as evaporative concentration (EC) are needed to enrich these compounds from matrices: Köke et al. [25] used mixed-bed SPE originally developed by Kern et al. [26] combining three SPE materials to enrich polar compounds from various environmental waters. The results were compared with a sample preparation method based on EC. The study showed that both approaches provided a higher coverage of analytes compared with Oasis HLB. Mean recoveries for the enriched analytes were similar; however, lower matrix effects were observed for the HLB method in HILIC-MS/MS. Mechelke et al. [27] compared EC with mixed-bed multilayer SPE for the enrichment of 590 organic substances from river water and wastewater. The results showed, that overall, EC was better suited for the enrichment of polar analytes (see also [3]), albeit considerable signal suppression was observed for the EC-enriched samples. However, there is still no method that covers the complete range of vPICs; therefore, further research is necessary to increase the analyte coverage and to further reduce matrix effects especially for polar analytes in biota samples, which may have more naturally occurring interferents.

SPE can be applied online and offline, with specific benefits and drawbacks of these two approaches. A major advantage of online SPE is that it analyzes the entire eluate from the SPE extract, hence providing better preconcentration factors, sensitivity, and recovery than most offline SPE approaches. In addition, online SPE has low solvent consumption requirements thereby decreasing the costs for organic solvents waste disposal [28]. Problems may arise from the compatibility with subsequent RPLC due to an insufficient elution strength [16]. However, Huntscha et al. [29] demonstrated that this problem can be overcome by adding water after the enrichment step to improve compatibility with the separation of polar compounds. In their study, a multiresidue method for the analysis of 88 polar organic micropollutants (with a broad range of physicochemical properties: log D (pH 7) ranging from − 4.2 to 4.2) was applied to analyze ground, surface, and wastewater using online mixed-bed multilayer SPE coupled to HPLC-MS. The majority of the compounds (~ 80%) could be quantified below 10 ng/l in groundwater and surface water and below 100 ng/l in wastewater using a sample volume of 20 ml. The method showed good relative recoveries.

Major drawbacks of SPE are e.g. limited sorption capacity and clogging of sorbent pores by suspended particles/matrix compounds. An alternative extraction mode that prevents these problems is dispersive SPE (d-SPE). It provides a large active surface for sorption. Recently, many novel materials were developed. Cai et al. [30] showed a successful application of vortex-assisted dispersive micro-SPE based on a novel porous metal organic framework for the determination of amphenicols and its metabolite in aquaculture water. Under optimal conditions, the relative recoveries were > 70%. Since d-SPE requires two centrifugation steps, automation of the technique is difficult, thus may not be suitable for higher throughput. A further development of d-SPE is magnetic SPE (m-SPE) using sorbents with superparamagnetic properties and high adsorption capacity, so that filtration or centrifugation steps can be avoided. Among the most recent functionalized magnetic materials are magnetite (Fe_3_O_4_) nanoparticles coated with silica, different polymers, nanomaterials, and ionic liquids [31]. In a study of Luo et al. [32], a magnetic composite made of graphene and Fe_3_O_4_ @ SiO_2_ was prepared by simple adsorption. It was used as an extraction medium for the effective and efficient enrichment of six sulfonamide antibiotics in environmental water samples. However, SPE cannot be regarded a green method given its relatively high solvent consumption.

Another mode of SPE is solid-phase microextraction (SPME). It is fast, versatile, sensitive, and solvent-free. The isolation and preconcentration of the analytes occurs in a single step, providing a simple sample preparation. In its most popular configuration, the SPME device consists of a fused-silica rod coated with a thin layer of a suitable polymeric coating (e.g., polydimethylsiloxane, polyacrylate, and carbowax). Since its introduction in 1990 by Pawliszyn [33], SPME applications significantly broadened also for the analysis of environmental samples [34–36]. Aresta et al. [37], for example, developed an SPME-LC-UV method for the determination of the polar antibiotic chloramphenicol in urine and environmental water samples. For SPME, polar carbowax fibers were used and provided sufficient extraction recoveries. However, SPME was shown to be less sensitive compared with SPE [38, 39]. Carbon nanotubes (CNTs) gained great interest [35], especially multi-walled CNTs with multiple layers of graphene [40] with their large surface-to-volume ratio and increased loadability. The extraction of non-polar, polar, and even ionic species is possible via both hydrophobic and ionic interactions [35], e.g., for the analysis of polar sulfonylurea herbicides in environmental water samples [41]. Recent SPME approaches also focused on a lower ecological impact [42, 43].

As well as SPME, microextraction by packed sorbent (MEPS) is an equivalent to SPE and can be expected to become a very promising sample preparation technique in the future for several reasons: it is fast and simple to use, it can be fully automated, it can cope with much smaller sample volumes than full-scale SPE (as small as 10 μl), which is of interest especially for the analysis of, e.g., plasma, urine, or biota samples like insects. MEPS sorbents can be used more than 100 times (even for complex samples). MEPS uses the same sorbents as conventional SPE in cartridges. Thus, downscaling of most existing SPE methods is possible. In MEPS, 1–4 mg sorbent is either inserted into the syringe (100–250 μl) as a plug or between the needle and the barrel in a cartridge. Sample extraction, enrichment, and clean-up are accomplished directly on the packed sorbent [44]. The number of extraction cycles can be increased by drawing and ejecting the sample through the needle into the syringe several times (draw-eject), leading to a higher recovery [45]. Another key aspect of MEPS is the small solvent volume used for the elution of the analytes, which makes it good choice for online coupling with LC [46] and CE [47] separations. Mostly, pharmaceuticals in wastewater were analyzed in environmental applications [48]. Morales-Cid et al. [47] demonstrated a new and innovative way to integrate MEPS into commercial CE equipment. This method provided automated sample clean-up and preconcentration from only a few microliters of sample, which is interesting in biota analysis. The robustness of the proposed technical implementation was demonstrated by the use of a (MEPS)-nonaqueous capillary electrophoresis (NACE)-MS method used to determine the very polar fluoroquinolones in urine. The detection limits (LODs) were in the range of 6.3–10.6 μg/l and absolute recoveries were in the range of 71–109%.

### Dispersive liquid-liquid microextraction

As a mode of liquid-liquid extraction (LLE), dispersive liquid-liquid microextraction (DLLME) was introduced by Rezaee et al. [49] for the extraction and preconcentration of organic analytes from water samples. A dispersed liquid phase is used to facilitate extraction from mostly small volumes (tens of μl range) of extraction solvent by fast equilibration due to the increased interfacial surface area between sample and extraction solvent. Many combinations with other sample preparation methods like SPE, single-drop microextraction, or DLLME itself allowed the purification and enrichment of a broad range of analytes (pharmaceuticals, pesticides, personal care products) in different samples (environmental samples, food, biological fluids) [50]. For hydrophilic compounds, recoveries have to be further improved. So far, the main strategy for the extraction of vPICs is compensating poor recoveries by high enrichment factors [51]. However, optimization strategies such as using ionic liquids as extraction solvents and ultrasound assistance in the extraction process led to the successful extraction of the polar fluoroquinolones (7 out of 8 with log *P* < 1) from groundwater samples [52]. In order to enable automated methods, there are new approaches to omit the dispenser solvent which could eliminate the centrifugation step and enable higher throughput [53].

### QuEChERS extraction

According to the current trend in analytical chemistry to develop environmentally friendly methods, QuEChERS extraction has become increasingly popular in many fields [54]. The extraction method was originally developed by Anastassiades in 2003 [55] to determine pesticides in fruits and vegetables. Since then, the method has been optimized and adjusted for different analytes and environmental matrices. For example, QuEChERS was used to extract pharmaceuticals from sewage and surface water [56], drinking water, treatment plant sludge [57], soil [58], invertebrates [59], and fish tissue [60, 61]. The method is based on LLE with mainly acetonitrile (partly also acetone or ethyl acetate) and water, often followed by a d-SPE for clean-up. Due to the miscibility of acetonitrile and water, a mixture of salts (NaCl and MgSO_4_) must be added to induce phase separation [55]. The ratio/volume of solvents and salts is used to optimize phase polarities and thus extraction efficiencies. For highly polar compounds, methanol can be added to enhance the recovery: for the quantification of quinolones and tetracyclines from fish tissue, an extraction solution based on a mixture of acetonitrile and methanol (75:25, v/v) resulted in relative recoveries ranging from 69 to 125% [62]. In contrast to food samples, the size and quantity of environmental biota samples is often limited. Therefore, the sample extraction techniques originally developed for food analysis have to be adapted. Often, miniaturization is necessary for invertebrate samples [59], e.g., carbamazepine and fluoxetine were quantified in single individuals of benthic invertebrates with relative recoveries > 85% [60]. Many non-polar and fatty compounds present in the mollusk matrix were removed using hexane as a third liquid phase during extraction. Overall, with its simplicity, environmental sustainability, and its compatibility with all relevant separation techniques, we suppose QuEChERS extraction to be increasingly applied in the future. However, we expect that not all vPICs can be extracted with acceptable efficiency.

### Electric field-driven sample preparation

There are new approaches using electric fields as auxiliary force to accelerate mass transfer of ionic analytes during extraction. Among them, electromembrane extraction (EME) has probably gained highest attention so far due to its simple setup. Introduced in 2006 by Pedersen-Bjergaard et al. [63], the usually aqueous sample and acceptor solutions are separated by a membrane, which can either be a supported liquid membrane (SLM) or a polymer-imprinted membrane. Application of an electric field across the two compartments results in migration of analytes according to their charge and electrophoretic mobility (both in the donor and acceptor solution but also in the membrane). This strategy has three benefits: (1) by using a smaller acceptor solution volume, enrichment of the analytes occurs together with (2) a clean-up from non-charged and oppositely charged matrix compounds, and (3) selectivity tuning by the membrane composition is possible (see below). This approach is only applicable for the extraction of ionizable and ionic compounds from various liquid samples or extracts. High matrix tolerance and compatibility with common analytical techniques like LC, GC, and CE have already been demonstrated [64]. Selectivity regarding the polarity of compounds can be tuned with different membrane compositions. In 2014, for example, Koruni et al. [65] presented the simultaneous extraction of both acidic and basic drugs over a broad range of polarities using two SLMs of different composition. However, further improvements to also target highly polar charged compounds are necessary and are addressed in current research [66]. In principle, EME is very environmentally friendly and potentially automatable, so we expect increased attention in the future, especially for water analysis. For extracts of biota or soil samples, this method is not (yet) established as mostly organic solvents are used for extraction. One possibility may be to evaporate the organic solvent and reconstituting the residue in water. Further research needs to address shortcomings such as low speed, low degree of automation, sensitivity, and recovery. Most studies so far used optimized methods for selected analytes; they have not yet been implemented for screening purposes.

## Trends in separation techniques

### Chromatographic techniques

In this chapter, we focus on trends in chromatographic techniques especially useful for the analysis of vPICs or for a broad analyte coverage in environmental samples, such as HILIC, SFC, and MMLC. Their advantages, disadvantages, and application to environmental samples are elaborated.

### Hydrophilic interaction liquid chromatography

In the last decade, HILIC-MS established itself as a valuable complementary approach to RPLC for the determination of highly hydrophilic, polar, and ionic compounds. This is also confirmed by the increase of HILIC applications [4] in various fields such as biology [67], food [68] and environmental science [24, 69]. Based on a combination of a polar stationary phase with a low aqueous and high organic content in the mobile phase, HILIC is able to improve chromatographic retention, resolution, and thus sensitivity for polar compounds [5], which may otherwise elute in or close to the void volume in RPLC. Further advantages over RPLC are higher applicable flow rates due the high organic content of the mobile phase and hence its lower viscosity. It is well suited for large volume injections of extracts with high organic content, often of interest in biota analysis. For the same reason, HILIC is also well compatible with online SPE as demonstrated by Fontanals et al. [16] with their first fully automated method based on online SPE coupled to HILIC-MS to determine a group of polar drugs and pharmaceuticals (log D (pH 7 ranging from − 1.8 to 1.38) in environmental water samples. The method had analyte recoveries near 100% and LODs ≤ 2 ng/l for most of the compounds. However, the high organic content of the mobile phase is a disadvantage when water samples are analyzed: the direct injection of large volumes of water as routinely done in RPLC (up to 100 μl) is not possible because of the high elution strength of water in HILIC [70]. Instead, dilution to ca. 80% acetonitrile is often required, significantly increasing LODs. In environmental analysis, HILIC was applied for the targeted analysis of polar herbicides [71], pesticides [72], and pharmaceuticals, such as antibiotics [17], drugs of abuse [16], cytostatics [73], antidiabetics [24, 74], and contrast agents [75, 76]. HILIC was successfully used as a complementary method to RPLC for the non-target screening of emerging polar organic compounds in wastewater [77]. To further extend the applicability of HILIC to a wider range of pharmaceutical compounds, various new HILIC stationary phases may be applied. Silica-based materials, in particular bare and amide-bonded silica, zwitterionic, and diol stationary phases, remain by far the most widely used stationary phases for HILIC in environmental applications with MS-compatible mobile phases [5]. In terms of green chemistry, HILIC is surely not advantageous due to the large amount of acetonitrile needed, necessitating a search for eco-friendlier alternatives. An example was given by dos Santos Pereira et al. [78], who demonstrated that biodegradable ethanol successfully replaced acetonitrile in the separation of vPICs. Another possibility to reduce the amount of organic solvents could be miniaturization of the analytical column, which is already a trend in bioanalysis [79]. However, this may be at the cost of a lowered sample loadability and is thus an option for biota but not for water analysis. Overall, we expect HILIC applications to increase as its polarity range almost ideally complements RPLC analysis (see Fig. 2), while the same equipment can be used. Multidimensional applications combining RPLC and HILIC are discussed in Section *2D applications*.

### Supercritical fluid chromatography

In recent years, SFC experienced a significant revival due to improvements in instrumentation, resulting in higher reliability and robustness [80]. Moreover, the advent of sub-2-μm particles and superficially porous particles in the stationary phases encouraged the use of UHPSFC. This gave rise to a further improvement of resolution and faster analyses, however, without the need for high pump pressure as encountered in ultra-high performance LC. Hyphenation to MS is increasingly used, which opened the way to new application fields such as bioanalysis, omics sciences, plant, food, and environmental analyses [80]. The mobile phase of modern SFC usually consists of compressed carbon dioxide, making it a “green method” [81]. Small volumes of modifiers such as methanol are added to tailor selectivity addressing different polarity ranges and optimize resolution. Regarding stationary phase chemistries, a large variety of stationary phases, from the most polar silica to the least polar well-endcapped or densely bonded alkyl-bonded silica, is now available, which enables to tailor selectivity [82].

**Fig. 3:**
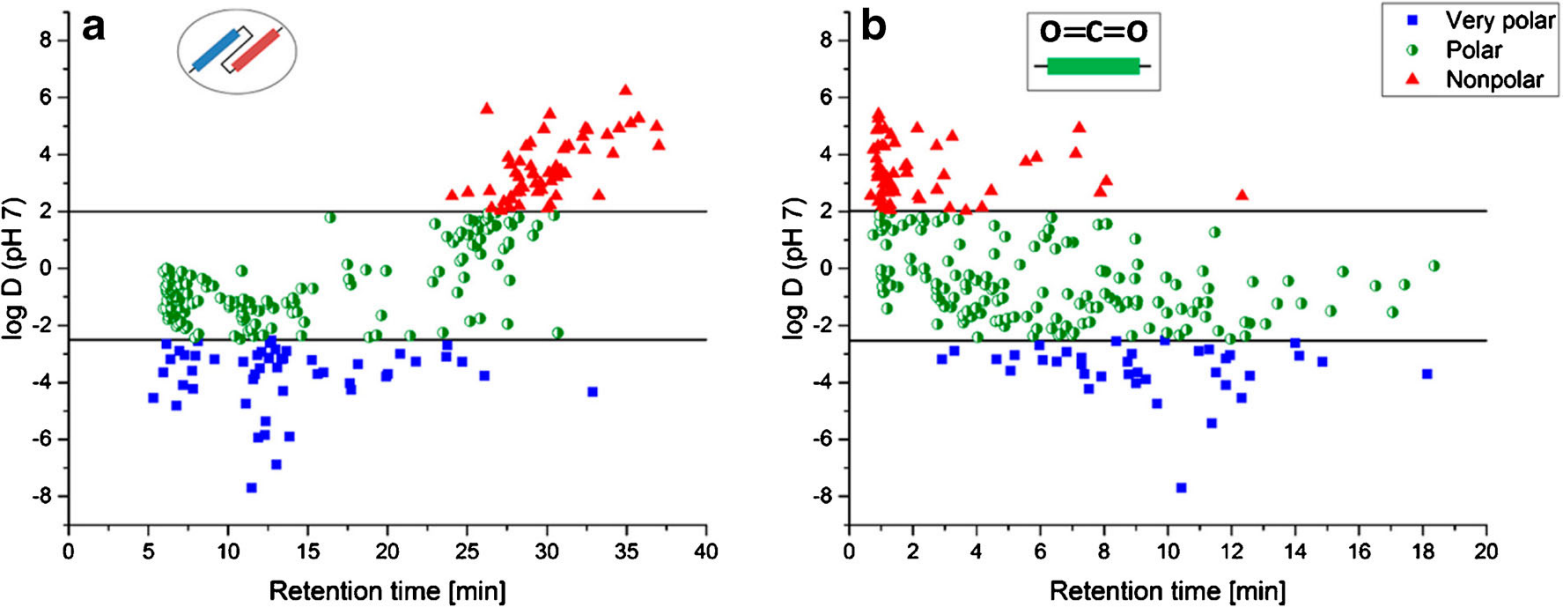
Plots of log D (pH 7) vs. retention time of standard compounds analyzed by RPLC-HILIC-TOF-MS (**a**) and SFC-TOF-MS (**b**). Very polar compounds (log D < − 2.5, blue rectangles) are mainly retained by HILIC in the RPLC-HILIC coupling, while non-polar compounds (log D > 2.0, red triangles) are exclusively retained by RPLC. Polar compounds are retained in both HILIC and RPLC, but retention in HILIC seems to be more likely with increased polarity. In SFC (**b**), non-polar compounds are retained less than very polar compounds. The retention patterns in the RPLC-HILIC coupling (**a**) show two groups that represent HILIC-(RT < 16 min) and RPLC-retained compounds (RT > 16 min). Compound log D increases with RT in RPLC, while the opposite occurs in HILIC. This retention behavior known from normal phase LC can partly be observed in SFC separations, too (**b**). Reprinted with permission from [81]. © 2020 American Chemical Society

In a seminal paper, two chromatographic separation strategies were compared, a serial RPLC-HILIC coupling vs. SFC [81]. For SFC, a zwitterionic HILIC column and a binary gradient of CO_2_ and 20 mM ammonium acetate in methanol (modifier) were used. Both systems were able to retain nearly all the 274 environmentally relevant compounds with very different polarities (log D (pH 7) ranging from − 7.71 to 7.67) providing a more comprehensive analysis than common RPLC-MS (see also Fig. 3). Such at least partially orthogonal methods can be used for cross-validation.

### Mixed-mode liquid chromatography

Mixed-mode stationary phases are gaining attention as more and more applications are published and new commercial columns appear on the market [83]. The main advantage of MMLC is that it provides more than one type of interaction between the stationary phase and the analytes, allowing the simultaneous determination of compounds with a wide range of physicochemical properties (e.g., ionic, basic, acidic and neutral chemicals, or hydrophilic and hydrophobic) in one run [84, 85]. The three most common types of MMLC are RP-ion exchange, HILIC-ion exchange, and RP-HILIC [86], which extends the application range of these methods but partially also combines their weaknesses and drawbacks [3]. Stationary phases are silica or polymer based; different hybrid and monolithic mixed-mode stationary phases are available [87]. The particle size of the current commercial sorbents is usually in the range of 3–7 μm and the stationary phases are synthesized on fully porous particles. In the near future, it can be expected that commercial sub-2-μm particles and superficially porous materials will be developed. This will lead to better separation efficiency and analysis time. Initially, mixed-mode columns were applied in bioanalysis, but their application was expanded to a variety of fields, including pharmaceutical analysis [88, 89], metabolism studies [90, 91], and recently also to environmental analysis [92–97]. González-Mariño et al. [97] described the first application of RP-ion exchange mixed-mode LC for the determination of drugs of abuse in wastewater. MMLC was shown to be a good alternative to traditional RPLC and HILIC for the separation of vPICs. Montes et al. [96] used a targeted MMLC-MS for the quantitative determination of 23 persistent and mobile organic contaminants (log D (pH 7) ranging from − 3.74 to 3.36) in surface and drinking water samples. In comparison with RPLC, MMLC proved superior in both retention and peak shape for ionic compounds, while also performing well for neutrals. In contrast to HILIC, the mobile phase of MMLC has a high aqueous content (as in RPLC); therefore, it is well suited for the direct injection of water samples. Further advantages of MMLC include the higher flexibility in adjusting separation selectivity and the possibility to use the mixed-mode sorbent in different elution modes [87]. MMLC can thus be an alternative to 2D applications. Besides many benefits, there are also some drawbacks related with these stationary phases. For example, when more than one type of interaction is active, asymmetric signals may evolve leading to reduced separation efficiency. Furthermore, method development has to consider the different separation modes and counteracting effects of optimization parameters may complicate this process. This necessitates compromises in separation performance, when analytes with a broad range of physicochemical characteristics are included. Additionally, Zahn et al. [3] indicated some limitations for RP-ion exchange columns, which are similar to the two retention mechanisms: poor retention of polar but neutral compounds and additionally the reliance on high electrolyte concentrations in the eluent to elute strongly retained ionic compounds which may cause detrimental effects in ESI [95]. However, with new mixed-mode stationary phases appearing on the market, MMLC-MS can be tailored to the polarity range of interest for specific questions and may be ideal for targeted or semi-non-target screening of classes of compounds.

### Ion chromatography

IC as a special mode of LC has already proven to be a powerful tool for the analysis of ionic compounds, mostly inorganic anions and cations in water samples and biological fluids [98] as well as small organic compounds in a broad range of sample types such as milk [99] or biological fluids [100], but also environmental samples [101]. For reliable IC analysis, e.g., of wastewater treatment effluents, sample preparation is often mandatory given the high matrix (salt) load of many environmental samples (see section *Sample preparation in environmental matrices*). Zakaria et al. [102] listed three major strategies for sample pretreatment: (1) samples may be purified by suitable ion exchange resins, e.g., silver cartridges for the removal of chloride [103]; (2) use of sensitive methods like inductively coupled plasma (ICP)-MS after strongly diluting the samples [104]; or (3) use of high capacity columns to increase loadability [105]. We see an additional possibility in the use of electric fields (see section *Electric field-driven sample preparation*) as both sample preparation and separation methods require charged analytes. An EME-IC system for the analysis of inorganic anions was presented [106]. Another option is the use of 2D-IC techniques [107] (see also section *2D applications*), as applied, e.g., for the analysis of bromate, chlorate, and five haloacetic acids in water [108]. Similar trends as in LC towards higher pressure and smaller particle sizes (see above) can be expected for IC to reach higher separation efficiencies and lower detection limits.

The successful analysis of acidic pharmaceuticals in biological fluids using IC with sensitive fluorescence detection after labeling was reported by Muhammad et al. [100]. A further increase in sensitivity is obtained when coupling IC with MS [109] upon further improvements of the interfaces. Recently, Stoll [110] summarized the state of the art of IC-MS. Limitations of current IC(-MS) instrumentation especially for (semi-)screening methods include the rather long column equilibration times and possible signal quenching by interactions of analytes with suppressors [111]. Publications in the field show a limited choice of suitable eluents as they must have a suitably high elution strength while being MS-compatible. Limitations for both ICP-MS and electrospray ionization (ESI)-MS were reported, describing decreased sensitivity in ICP-MS by using carbonate/bicarbonate eluents without a suppressor as a result of contamination of skimmer cones [112] and a general signal suppression in ESI due to high concentrations of eluents [113]. Overall, improvements with optimized elution buffers [113] as well as instrumentation and system design [114] are still subject to current research. Another approach is the reduction of the column inner diameters to, e.g., 0.4 mm [115], or to use an organic make-up solvent such as acetonitrile or isopropanol [116] to increase MS sensitivity.

The two major MS interfaces used for IC are ICP and ESI. Whereas IC-ICP-MS is suitable for elemental analysis in a broad range of different samples including, e.g., organic compounds with halogen substituents, IC-ESI-MS enables to analyze low molecular mass organic compounds. The general advantages of ICP-MS for different environmentally relevant pollutant species (e.g., pharmaceuticals, flame retardants, pesticides,…) were worked out nicely by Pfröfrock and Prange [117]. With ICP-MS, the detection and selective screening of different molecules containing covalently bound (hetero-)elements such as phosphorus, arsenic, or halogens is possible. Sacher et al. [118], for example, developed an IC-ICP-MS method for iodinated X-ray contrast agents in surface water. Whereas a full non-target screening cannot be reached with IC-ICP-MS, it may be possible to target sulfur species in a screening approach, interesting to study the transformation of pharmaceuticals and pesticides to sulfur- or phosphor-containing species, e.g., metolachlor with charged transformation products [119]. In addition, quantification is possible without internal standards, if the stoichiometry is known [120, 121]. Especially the complementary combination of ICP-MS for screening and ESI-MS for identification is an interesting approach [122, 123], which will likely be applied more often in the future.

Speciation analysis by IC-ICP-MS for environmental analysis was summarized in a book edited by Michalski [124]. For biota, the potential of IC-ICP-MS has already been exploited for the detection of arsenic and selenium species in fish tissue by Reyes et al. [125] but to our knowledge not yet for pharmaceuticals. IC-ICP-MS with its high selectivity will be of future interest especially for targeted or selective screening approaches in samples of high matrix load such as biota samples. Generally, this can enable a broader screening of vPICs and their transformation products in biota, especially for compounds with iodine or bromine, which are rare in biological samples.

Successful applications of IC-ESI-MS for the analysis of environmental samples show the increasing interest for the analysis of pesticides in food samples. Polar pesticides being charged over a broad pH range like glyphosate, ethephon [113], or paraquat [126] were successfully analyzed in food samples by IC-ESI-MS/MS. In environmental samples, this technique is mostly applied for the analysis of inorganic anions and cations as well as small organic acids [127]. However, in recent years, there has been progress in the analysis of polar pesticides in environmental waters by IC-MS. Glyphosate and two of its metabolites (all log *P* < − 2) were successfully analyzed in surface and drinking waters reaching LODs in the low ng/l range [128]. The analysis of haloacetic acids in drinking water was also successful [129], which is very fortunate given the new regulations in the European Union (Water Framework Directive, WFD, 2000/60/EC) requiring the sensitive analysis of these compounds. We expect further applications for the analysis of wastewater micropollutants and semi-targeted screenings, as they already proved valuable in forensic analysis of gunshot residues [130].

### Electromigrative separation techniques

Electromigrative separation techniques like CE are an alternative to IC for the analysis of ionic or ionizable micropollutants. The general applicability and potential of CE in environmental analysis was shown in numerous applications of pesticide analysis summarized in recent reviews [131, 132]. The use and state of the art of CE analysis of various pharmaceuticals in environmental samples was described by Hamdan in 2017 [133].

Whereas CE is already well established for the analysis of small molecules in human body fluids [134], the use of electromigration separation techniques for the analysis of vPICs in environmental samples is still challenging. The loadability for the commonly used capillaries of only 50 μm inner diameter is limited and often gives rise to relatively high detection limits. This can be overcome improving sample preparation and enrichment methods dedicated to CE, e.g., sample preparation using online or offline SP(M)E (see Hamdan [133]). Online enrichment is possible via large volume sample stacking [135], field-amplified sample injection [136], and (transient) isotachophoresis [137, 138]. Offline electro-driven enrichment techniques like EME are gaining more and more popularity [139] and their hyphenation with CE for the analysis of environmental samples was reported [140] (see also section *Electric field-driven sample preparation*). There are optimized CE-diode array detection (DAD) methods which reach detection limits in the medium ng/l range [141]; however, these are rather exceptional. More often, MS [142] and sometimes fluorescence detection [143], mostly after derivatization [144], are used.

As for LC, MS is the detection method of choice for non-target screenings, but today, most CE-MS methods are still established in research laboratories only, although (1) there are less restrictions compared with IC-MS hyphenation (e.g., eluent composition) and (2) ESI-MS-interfaces compatible with both LC and CE separation systems are available. In addition, solvent consumption is low compared with chromatographic techniques, which makes CE and CE-MS interesting as “green” alternatives. Although many CE(-MS) methods barely reach detection limits below 1 μg/l [133], there are already promising approaches combining CE-MS with different sample preparation methods [145, 146]. Additionally, an enrichment-free approach was recently published by Höcker et al. [147] enabling the analysis of anionic micropollutants like haloacetic acids and halomethanesulfonic acids in drinking water, reaching LOQs between 30 and 500 ng/l.

CE was shown to be well suited for the analysis of biological fluids, but there are only few examples for its use in biota analysis: Deng et al. [148] analyzed the polar antibiotic tetracycline in crucian carp muscle using electrochemiluminescence detection. Sun et al. [149] analyzed sulfonamides in shrimp, sardine, and anchovy with CE-DAD. Both methods reached detection limits in the μg/l range. Coupling CE to sensitive MS detection even led to detection limits in the upper ng/l (low ng/g) range for the analysis of the antidiabetic drug metformin in fish without sample preconcentration [150], requiring only small sample volumes, which enabled analyzing individual fish organs.

### 2D applications

The aims of 2D approaches are: (1) maximum sample information, the comprehensive analysis of micropollutants over a broad range of physicochemical characteristics (today mostly polarity) for non-target analysis. For this, RPLC-HILIC has already been established by Bieber et al. [81]. Similar approaches are well known in peptide analysis [151]. (2) With 2D techniques, peak capacity can be increased using ideally orthogonal separation selectivity in the second dimension. The two separation dimensions can either be coupled consecutively or by heart-cutting selected zones to enhance resolution and minimize matrix effects, e.g., comprehensive couplings of IC×RPLC [152] or IC×CE [153]. Chromatographic and electromigration separations were successfully combined in bioanalysis [154]. (3) The first dimension may also be used as a clean-up step as shown for IC-IC by Zakaria et al. [102] using the first dimension to eliminate inorganic salt components prior to the quantification of bromate in sea water samples. Although LODs of only 60 μg/l of bromate were obtained, this approach might also be interesting for the analysis of pharmaceuticals in environmental samples containing high concentrations of salt using IC with sensitive MS detection. Possibly, additional preconcentration and desalting steps, e.g., using SPE, are necessary to meet the required detection limits.

It has to be noted that comprehensive applications are generally limited by the first dimension with regard to polarity or charge. Improved instrumental setups for hyphenation and testing some of the new materials available today (e.g., MMLC stationary phases, see section *Mixed-mode liquid chromatography*) can result in increased matrix tolerance and higher sensitivity for complex samples. Stevenson et al. [155] developed an offline 2D-LC separation of a β-lactoglobulin tryptic digest with the same mixed-mode stationary phase in both dimensions. In the first dimension, the mobile phase pH was 7, while it was adjusted to pH 2 in the second dimension evoking different separation mechanisms. Greater separation efficiency was observed for the mixed-mode column in comparison with classical C18, thereby providing larger peak capacity than the C18 column. Similar applications will be of interest in environmental analysis. With the advent of commercial equipment for 2D applications, its application in environmental analysis will surely rise, though merely for research than for routine analysis. A major limitation is the data evaluation, where further software developments are required.

### Summary on future perspectives

The impressive development of new analytical methods enabled the analysis of many vPICs in environmental samples. These developments comprise new materials, instrumentation, miniaturization, and automation both for sample preparation and separation. In this article, we showed that some current trends are partly due to a revival of methods such as SFC, partly due to the use of developments from other fields such as QuEChERS extraction from food science, and MMLC from pharmaceutical analysis or some 2D applications from bioanalysis. In the following, we want to summarize future perspectives.

Due to the high demand for improved limits of detection and selectivity, many new SPE materials for vPICs were commercialized but none of the sorbents available can cover the whole range of compounds. Especially, the retention of very polar analytes is still an issue. Both mixed-bed SPE and EC were shown to also cover very polar compounds. However, only a few mixed-bed cartridges are commercially available and EC does not include a sample clean-up. New materials like carbon-based nanomaterials can be used to improve the extraction efficiency but they are mostly suitable only for target analysis or specialized applications.

For ionic and ionizable compounds, we expect electro-based enrichment techniques to find applications beyond inorganic ions [106], especially for the analysis of ionic or ionizable pharmaceuticals in environmental samples. For electro-based enrichment techniques, further effort is required to achieve an acceptable degree of sample throughput by automation and miniaturization for both monitoring strategies and (eco)toxicological research. For matrix removal, these developments are believed to be prioritized compared with online sample pretreatment and 2D approaches.

We are convinced that HILIC will complement RPLC routine analysis as the expertise for liquid chromatography is high and implementation is straightforward. Future research will show which stationary phases will become the gold standard in the future. SFC research is very active and due to the development of modern instrumentation, reproducible results are obtained today. In the pharmaceutical industry, SFC can already replace RPLC as a routine technique for the separation of chiral substances on a preparative scale [156]. In environmental analysis, the actual potential of SFC can only be estimated, since applications are still rare. However, it is anticipated that SFC may partly replace RPLC, as a wider polarity range is covered, even when compared with 2D techniques such as RPLC-HILIC. SFC may then become a suitable tool also for monitoring campaigns. Thus, it will be interesting to see if 2D techniques will find more application in environmental analysis as is currently seen in bioanalysis, especially peptide analysis. While instrumental developments led to the commercial availability of dedicated instrumentation for comprehensive 2D-LC, software tools still show limitations. Possibly, MMLC will open up new possibilities in 2D separation methods, when used as a first dimension with a broad analyte coverage. We see a great potential in MMLC since it improves the separation of vPICs (both basic and acidic) while also retaining neutral chemicals. MMLC thus combines the benefits of different chromatographic modes. Manufacturers of stationary phases also reacted with increased production of MMLC stationary phases.

Finally, attractive alternatives for ionizable compounds, also with regard to “green chemistry,” are IC-MS and CE-MS. However, environmental CE-MS applications afford more research for analyte enrichment and IC-MS applications for improved stationary phases for a greater choice of MS-compatible eluents. It is difficult to judge if CE-MS with a lower instrumental requirement than IC-MS will be implemented in laboratories dominated by chromatographic expertise.

In our opinion, the gap described in 2016 by Reemtsma et al. [2] for the analysis of persistent and mobile organic compounds still exists. With the latest developments in analytical chemistry, filling this gap seems feasible. However, most methods which are well suited have not yet left laboratory scale applications so that their applicability in monitoring campaigns remains to be shown. Establishing new analytical processes in general is a time-consuming process for laboratories. Acceptance by the community and regulatory bodies is another hindrance. Often, modified or optimized established methods are accepted more quickly and are easier to implement with regard to existing instrumentation and expertise. However, with the slow implementation of new methodology, chances are missed to increase efficiency, scope, matrix tolerance, environmental friendliness, etc.

All in all, however, we have to keep in mind that no method is capable to fulfill all requirements from environmental science and (eco)toxicology given the wealth of compounds with their broad range of physicochemical characteristics. New strategies have to find compromises between different needs, e.g., analyte coverage, matrix compatibility, or analysis time and costs. Additionally, other drivers like automation, miniaturization in biota analysis, and organic solvent consumption have to be considered for current and future developments for the analysis of vPICs.

## Electronic supplementary material

ESM 1 (PDF 380 kb)
