# Supplementary material for: Trends in sample preparation and separation methods for the analysis of very polar and ionic compounds in environmental water and biota samples
Source: Anal Bioanal Chem. 2020 Jul 24;412(24):6149–65. doi: 10.1007/s00216-020-02811-5 (PMC7442764; doi:10.1007/s00216-020-02811-5)
Supplement: Supplementary file 1 — (PDF 380 kb) [file 216_2020_2811_MOESM1_ESM.pdf]

Analytical and Bioanalytical Chemistry

Electronic Supplementary Material

Trends in sample preparation and separation methods for the analysis of very polar and ionic compounds in environmental water and biota samples

Sarah Knoll, Tobias Rösch, Carolin Huhn

This Supporting Information contains a table presenting a non-comprehensive, alphabetically ordered list of 237 compounds with log P values ≤ 1 analyzed in 63 cited articles. All properties (pK_a values, log P and log D values and charge state) were calculated with Chemicalize provided by ChemAxon. The column "function" contains one of the main uses from which the pie chart in Fig. 1B was generated.

Table 1 Non-comprehensive, alphabetically ordered list of 237 compounds with log P values ≤ 1 analyzed in 63 here cited articles. All properties (pK_a values, log P and log D values and charge tate at pH 7.4) were calculated with Chemicalize provided by ChemAxon

analyte	strongest		log P	log D (pH)			charge pH 7.4	function
	acidic pKa	basic pK_a		1.7	7.4	8.0		
2′,2′-difluoro-deoxyuridine	9.89		-1.08	-1.08	-1.08	-1.08	0	human metabolites
2-phenyl-5-benzimidazole-sulfonic acid	-2.18	4.93	-0.14	-0.14	0.09	0.09	-1.00	UV filter
2-phenylbenz-imidazole-5-sulphonic acid	-2.18	4.93	-0.14	-0.14	0.09	0.09	-1.00	UV filter
3,4-dihydroxyphenylacetic acid	3.61		1.00	1.00	-2.34	-2.49	-1.01	human metabolites
3-methylphosphinico-propionic acid	2.00		-1.29	-1.47	-6.23	-6.71	-2.00	pesticide metabolite
4-acetamido-antipyrine	12.52	-0.76	0.15	0.15	0.15	0.15	0	drug metabolites
4-formylamino-antipyrine	12.66	-0.75	0.11	0.11	0.11	0.11	0	drug metabolites
4-methylamino-antipyrine		1.24	0.77	0.64	0.77	0.77	0	drug metabolites

analyte	strongest		log P	log D (pH)			charge pH 7.4	function
	acidic pKa	basic pK_a		1.7	7.4	8.0		
4-methylthiosemi-carbazide	14.10	3.85	-0.55	-2.66	-0.55	-0.55	0	intermediate compound
5-fluorouracil	7.18		-0.66	-0.66	-1.08	-1.51	-0.63	cytostatics
6-acetylmorphine	10.15	8.96	0.91	-2.27	-0.39	0.17	0.97	drug metabolite
abacavir	15.43	5.80	0.39	-1.59	0.38	0.38	0.19	Nucleoside Reverse Transcriptase Inhibitors
abacavir carboxylate	3.77	5.80	-1.18	-1.29	-2.48	-2.72	-0.81	drug metabolite
acephate	10.54		-0.33	-0.33	-0.33	-0.33	0	insecticide
acesulfame	3.02		-0.55	-0.57	-1.49	-1.49	-1.00	artificial sweetener
acetaminophen	9.46		0.91	0.91	0.91	0.91	0	Analgesics and antipyretics
acetic acid	4.54		-0.22	-0.22	-3.00	-3.41	-1.00	intermediate compound
acrilyamide	0		-0.27	-0.27	-0.27	-0.27	0	intermediate compound
acyclovir	11.98	3.02	-1.03	-2.1	-1.03	-1.03	0	antiviral
amidosulfuron	3.21	2.49	-1.19	-1.77	-1.70	-1.7	-1.00	herbicide
amikacin	12.16	9.61	-8.58	-20.72	-15.10	-12.91	3.80	aminoglycoside antibiotics
aminopyralid	1.11	4.97	0.27	0.2	-1.84	-2.04	-1.00	herbicide
amoxicillin	3.23	7.22	-2.31	-3.02	-2.67	-3.04	-0.61	β-lactam antibiotic
AMP[1]	1.23	3.92	-4.75	-4.53	-5.75	-6.26	-1.96	nucleotide
AMPA[2]	1.90	9.36	-2.33	-2.52	-3.23	-4.33	-1.03	pesticide metabolite
ampicillin	3.24	7.23	-2.00	-2.72	-2.36	-2.72	-0.6	penicillin
ascorbate	4.16		-1.26	-2.12	-4.81	-4.87	-1.05	vitamin
aspartame	3.53	8.53	-2.22	-2.96	-2.25	-2.32	-0.07	artificial sweetener
atenolol	14.08	9.67	0.43	-2.82	-1.80	-1.24	0.99	beta blocker

analyte	strongest		log P	log D (pH)			charge pH 7.4	function
	acidic pKa	basic pK_a		1.7	7.4	8.0		
atrazine-hydroxydesethyl	12.63	5.62	1.00	-0.83	1.01	1.01	0	pesticide metabolite
azide	5.09		0.08	-0.04	-0.15	-0.15	-1.00	intermediate compound
barbitone	7.48		0.72	0.72	0.46	0.1	-0.45	hypnotic
bentazon	2.03		0.76	0.61	-0.19	-0.19	-1.00	herbicide
benzene-sulfonamide	10.24		0.58	0.58	0.58	0.58	0	intermediate compound
benzoylecgonine	3.15	9.54	-0.60	-1.30	-0.60	-0.60	-0.01	drug metabolites
bromate	1.27		0.19	-0.37	-2.19	-2.19	-1.00	inorganic anion
bromide	-8.00		0.80	1.02	1.02	1.02	-1.00	inorganic anion
bromochloro-acetic acid	1.97		0.79	0.60	-2.80	-2.80	-1.00	disinfection byproduct
bromomethane-sulfonic acid	-2.07		0.00	-2.36	-2.38	-2.38	-1.00	disinfection byproduct
butyric acid	4.91		0.92	0.92	-1.53	-2.04	-1.00	industrial chemical
caffeine		-1.16	-0.55	-0.55	-0.55	-0.55	0	psychoactive drug
cAMP[3]	1.83	3.94	-3.40	-3.32	-3.69	-3.69	-1.00	second messenger
carbonate	3.50		0.25	0.24	-3.13	-3.24	-1.00	other
cCMP[4]	1.75	4.18	-2.01	-2.29	-4.33	-4.38	-1.00	second messenger
cefadroxil	3.25	7.22	-2.45	-3.16	-2.81	-3.17	-0.61	β-lactam antibiotic
cefoperazone	3.19	-1.98	-0.90	-0.91	-4.35	-4.42	-1.01	β-lactam antibiotic
cefotaxime	2.73	3.58	-1.49	-2.02	-4.2	-4.24	-1	β-lactam antibiotic
cefradin	3.27	7.6	-2.45	-3.16	-2.64	-2.93	-0.39	β-lactam antibiotic
ceftiofur	2.52	3.49	0.11	-0.27	-2.46	-2.49	-1	β-lactam antibiotic
cefuroxime	2.96	-1.16	-0.90	-0.93	-4.38	-4.42	-1	β-lactam antibiotic
cephalexin	3.26	7.23	-2.14	-2.86	-2.5	-2.85	-0.6	β-lactam antibiotic

analyte	strongest		log P	log D (pH)			charge pH 7.4	function
	acidic pKa	basic pK_a		1.7	7.4	8.0		
cephalosporin C	1.83	9.22	-4.39	-4.54	-7.64	-7.77	-1.01	β-lactam antibiotic
cephapirin	3.35	4.99	-2.05	-1.94	-4.17	-4.38	-1.00	β-lactam antibiotic
cephazolin	2.84	0.26	-1.52	-1.57	-5.01	-5.04	-1.00	β-lactam antibiotic
cGMP[5]	1.79	2.90	-2.10	-2.19	-4.31	-4.31	-1.00	second messenger
chloramphenicol	8.69		0.88	0.88	0.86	0.81	-0.05	broadband antibiotic
chlorate	4.62		0.04	0.04	-2.19	-2.29	-1.00	inorganic anion
chloride	-7.00		0.61	0.83	0.83	0.83	-1.00	inorganic anion
chlorite	-4.57		0.18	-2.2	-2.2	-2.2	-1.00	inorganic anion
chlormequat			-3.31	-3.31	-3.31	-3.31	-1.00	pesticide
chloromethane-sulfonic acid	-2.30		-0.19	-2.55	-2.56	-2.56	-1.00	disinfection byproduct
chlortetracycline	6.99	6.18	-2.90	-3.58	-3.45	-4.04	-0.83	tetracycline antibiotic
choline	13.97		-4.66	-4.66	-4.66	-4.66	1-1.00	essential nutrient
chromate	-2.30		-3.71	-9.2	-8.23	-8.23	-2.00	inorganic anion
ciprofloxacin	5.56	8.77	-0.86	-1.69	-0.87	-0.91	-0.01	fluoro-quinolone
citrate	3.05		-1.32	-1.34	-9.47	-10.54	-2.99	organic acid
clindamycin-sulfoxide	12.29	7.46	-0.98	-4.48	-1.31	-1.09	0.54	drug metabolites
CMP[6]	1.79	2.90	-2.10	-2.19	-4.31	-4.31	-1.00	nucleotide
cyanate	-1.29		-0.54	-3.23	-3.54	-3.54	-1.00	other
cyanuric acid	5.55		0.98	0.98	0.98	0.98	0	intermediate compound
cyclamate	-0.83		0.61	-1.54	-1.77	-1.77	-1.00	artificial sweetener
cytarabine	12.55	4.19	-2.80	-4.65	-2.8	-2.8	0	cytostatics
danofloxacin	5.49	7.31	0.06	-1.56	-0.15	-0.55	-0.53	fluoro-quinolone
desmethyl rantinidine		8.4	0.61	-2.66	-0.43	0.06	0.91	drug metabolite
dibromoacetic acid	1.60		0.70	0.35	-2.89	-2.89	-1.00	disinfection byproduct

analyte	strongest		log P	log D (pH)			charge pH 7.4	function
	acidic pKa	basic pK_a		1.7	7.4	8.0		
dibromobutyric acid	2.77		0.87	0.84	-2.68	-2.71	-1	disinfection byproduct
dibromomethanesu lfonic acid	-2.38		0.96	-1.34	-1.34	-1.34	-1	disinfection byproduct
dibromopropionic acid	2.45		0.80	0.73	-2.77	-2.78	-1	disinfection byproduct
diethanol amine	15.30	9.26	-1.57	-4.82	-3.42	-2.85	0.99	intermediate compound
diethyl amine		10.58	0.52	-2.72	-2.39	-1.98	1	intermediate compound
diethyl phosphate	1.95		0.45	0.26	-1.93	-1.93	-1	other
difluoroacetic acid	2.00		0.19	0.01	-3.33	-3.34	-1	disinfection byproduct
diisopropanol amine	15.00	9.55	-0.74	-3.98	-2.86	-2.3	0.99	intermediate compound
dimethyl amine		10.52	-0.19	-3.43	-3.07	-2.64	1.00	intermediate compound
diquat			-7.03	-7.03	-7.03	-7.03	2.00	herbicide
doxycycline	7.33	5.82	-3.34	-4.10	-3.65	-4.14	-0.63	antibiotic
DTPA-BMA-Gd	1.02	8.34	-8.74	-8.94	-12.47	-13.1	-1.94	
emitricitabine S-oxide	14.01	1.41	-2.27	-2.45	-2.27	-2.27	0	pesticide metabolite
emtricitabine	14.29	1.74	-0.90	-1.21	-0.90	-0.90	0	nucleoside reverse-transcriptase inhibitor (NRTI)
emtricitabine carboxylate	3.31	1.54	-0.72	-0.81	-4.00	-4.08	-1.00	drug metabolite
enalapril	3.67	5.20	0.59	-0.79	-1.06	-1.22	-0.99	ACE inhibitor
enrofloxacin	5.55	7.24	0.51	-1.21	0.27	-0.14	-0.57	fluoro-quinolone
ethephon	1.79		-0.57	-0.82	-2.93	-3.05	-1.21	plant growth regulator
ethyl glucuronide	3.45		-1.61	-1.62	-4.54	-5.00	-1.00	human metabolites
ethyl sulfate	-2.08		-0.11	-2.47	-2.48	-2.48	-1	intermediate compound

analyte	strongest		log P	log D (pH)			charge pH 7.4	function
	acidic pKa	basic pK_a		1.7	7.4	8.0		
ethylenthiourea	13.88	6.41	-0.28	-3.23	-0.11	-0.08	0.19	intermediate compound
flonicamid	12.56	3.37	0.24	-0.67	0.24	0.24	0	insecticide
florfenicol	8.49		0.67	0.67	0.64	0.56	-0.08	broadband antibiotic
florfenicol-amine	13.64	8.05	-0.43	-3.46	-1.16	-0.76	0.82	broadband antibiotic
fluconazole	12.68	2.30	0.56	0.56	0.56	0.56	0	antifungal medication
fluoride	3.17		0.15	0.16	0.37	0.37	-1.00	inorganic anion
formate	4.27		-0.27	-0.27	-3.26	-3.59	-1.00	organic acid
fosetyl-Al		-1.85	0.16	0.16	0.16	0.16	0	fungicide
fructose	10.28		-2.76	-2.76	-2.76	-2.76	0	carbohydrates
fumarate	3.35		-0.04	-0.05	-6.51	-6.89	-2.00	food additive
gabapentin	4.63	9.91	-1.27	-2.04	-1.27	-1.28	0	anticonvulsant, antiepileptic
gadodiamide	-6.62	-0.30	-16.15	-15.35	-14.62	-14.78	1.33	contrast agent
Gd-BOPTA	1.74	8.9	-4.33	-5.06	-16.03	-16.93	-3.69	contrast agent
Gd-BT-DO3A/Gadovist	1.24	7.77	-8.55	-9.32	-12.18	-13.04	-2.18	contrast agent
Gd-DOTA	1.16	7.48	-6.74	-6.99	-14.1	-15.19	-3.37	contrast agent
Gd-DTPA	-0.98	8.80	-5.94	-6.45	-17.9	-18.82	-3.90	contrast agent
gemcitabine	11.52	3.65	-1.47	-3.13	-1.47	-1.47	0	cytostatics
gentamicin	12.55	10.12	-3.14	-18.72	-11.33	-8.83	4.52	aminoglycoside antibiotic
glufosinate	1.86	9.53	-3.46	-3.58	-6.67	-6.7	-1.01	herbicide
glycolic acid	3.53		-1.04	-1.05	-4.41	-4.52	-1.00	other
glyphosate	-0.58	9.56	-3.10	-3.11	-7.26	-7.79	-1.89	herbicide
GMP[7]	1.23	2.84	-3.12	-3.42	-6.36	-6.87	-1.97	nucleotide
guanylurea	13.62	9.79	-1.77	-4.05	-3.82	-3.48	1.00	drug metabolite
hydrochloro-thiazide	9.09		-0.58	-0.58	-0.58	-0.61	-0.02	diuretics
iodate	0.85		0.18	-0.72	-2.2	-2.2	-1.00	inorganic anion

analyte	strongest		log P	log D (pH)			charge pH 7.4	function
	acidic pKa	basic pK_a		1.7	7.4	8.0		
iodixanol	11.44	-1.06	-2.06	-2.06	-2.06	-2.06	0	contrast agent
iohexol	11.73	-1.36	-1.95	-1.95	-1.95	-1.95	0	contrast agent
iomeprol	11.73	-1.36	-1.45	-1.45	-1.45	-1.45	0	contrast agent
iopamidol	11.00	-1.55	-0.74	-0.74	-0.74	-0.74	0	contrast agent
iopentol	11.73	-1.36	-1.31	-1.31	-1.31	-1.31	0	contrast agent
iopromide	11.09	-1.40	-0.44	-0.45	-0.44	-0.45	0	contrast agent
ioversol	11.72	-1.36	-2.14	-2.14	-2.14	-2.14	0	contrast agent
ipratropium	15.15		-1.82	-1.82	-1.82	-1.82	1.00	bronchodilators
itaconate	3.56		0.05	0.05	-6.25	-6.73	-2.00	intermediate compound
kanamycin	12.05	9.34	-7.06	-19.19	-12.2	-10.28	3.5	aminoglycoside antibiotic
lactate	3.78		-0.47	-0.48	-3.74	-3.92	-1	human metabolites
lamivudine	14.29	4.30	-1.10	-2.97	-1.10	-1.10	0	nucleoside reverse transcriptase inhibitors
l-carnitine	4.20		-4.88	-4.88	-4.12	-4.12	0	human metabolites
levofloxacin-ofloxacin	5.35	6.72	0.09	-1.93	-0.51	-1.02	-0.80	fluoro-quinolone
lomefloxacin	5.45	8.78	-0.43	-1.24	-0.43	-0.47	-0.02	fluoro-quinolone
maleate	2.85		-0.04	-0.07	-5.21	-5.82	-1.99	organic acid
malonate	2.43		-0.33	-0.41	-5.34	-5.93	-1.98	organic acid
maltose	11.25		-4.70	-4.70	-4.70	-4.70	0	carbohydrate
maltotriose	11.22		-6.47	-6.47	-6.47	-6.47	0	carbohydrates
marbofloxacin	5.28	6.69	-0.61	-2.59	-1.24	-1.74	-0.82	fluoro-quinolone
melamine		9.56	-0.60	-3.58	-2.34	-2.02	0.99	intermediate compound

analyte	strongest		log P	log D (pH)			charge pH 7.4	function
	acidic pKa	basic pK_a		1.7	7.4	8.0		
mepiquat			-3.12	-3.12	-3.12	-3.12	1.00	plant growth regulator
metformin		12.3	-0.92	-5.75	-5.62	-5.37	2.00	antidiabetic
methane-sulfonic acid	-1.61		-0.96	-3.29	-3.34	-3.34	-1.00	intermediate compound
methylisothiazolin e	4.54		0.93	-1.81	0.93	0.93	0	intermediate compound
metronidazole	15.41	3.03	-0.46	-1.36	-0.46	-0.46	0	nitroimidazole antibiotics
monobromo-acetic acid	2.64		0.50	0.45	-3.01	-3.02	-1.00	disinfection byproduct
monobromo-propionic acid	3.24		0.74	0.72	-2.7	-2.77	-1.00	disinfection byproduct
monochloro-acetic acid	3.06		0.31	0.30	-3.15	-3.20	-1.00	disinfection byproduct
monochloro-butyric acid	4.04		0.84	0.84	-2.29	-2.55	-1.00	disinfection byproduct
monochloro-propionic acid	3.72		0.55	0.55	-2.74	-2.91	-1	disinfection byproduct
monoethanol amine	14.84	9.37	-0.98	-4.01	-2.92	-2.36	0.99	intermediate compound
monoethyl amine		10.23	-0.27	-3.30	-2.89	-2.43	1.00	intermediate compound
monoethyl phosphate	1.80		-0.29	-0.54	-3.33	-3.84	-1.88	intermediate compound
monofluoro-acetic acid	3.13		-0.15	-0.17	-3.61	-3.66	-1.00	disinfection byproduct
monoisopropanol amine	14.47	9.62	-0.73	-3.76	-2.89	-2.34	0.99	intermediate compound
monomethyl amine		10.08	-0.63	-3.66	-3.15	-2.66	1.00	intermediate compound
mono-n-butyl phosphoric acid	1.81		0.68	0.43	-2.36	-2.86	-1.88	intermediate compound
morphine	10.26	9.12	0.90	-2.3	-0.60	-0.03	0.98	opioid
morpholine		8.51	-0.41	-3.65	-1.55	-1.03	0.93	intermediate compound
moxifloxacin	5.49	9.51	-0.51	-1.28	-0.50	-0.51	0.02	fluoroquinolone

analyte	strongest		log P	log D (pH)			charge pH 7.4	function
	acidic pKa	basic pK_a		1.7	7.4	8.0		
N,*N*-dimethylsulfamide			-0.63	-0.63	-0.63	-0.63		herbicide metabolite
N^4-acetylsulfa-merazine	6.88	-1.81	0.59	0.59	0.09	-0.17	-0.77	drug metabolite
N^4-acetylsulfa-methoxazole	5.88	0.38	0.86	0.84	0	-0.06	-0.97	drug metabolite
nadolol	13.59	9.76	0.87	-2.38	-1.44	-0.89	1.00	beta blocker
nalidixic acid	5.77	4.66	0.79	-0.68	-0.45	-1.01	-0.90	fluoro-quinolones
n-butyl amine		10.21	0.70	-2.33	-1.91	-1.45	1.00	intermediate compound
neohesperidin dihydrochalcone	8.80		0.75	0.75	0.73	0.68	-0.05	artificial sweetener
neomycin	12.15	9.71	-8.42	-26.61	-15.47	-12.65	5.09	aminoglycoside a ntibiotic
nitrate	-1.40		-0.20	-2.57	-2.57	-2.57	-1.00	inorganic anion
nitrite	3.39		0.17	0.17	0.17	0.17	-1.00	inorganic anion
norephedrine	13.9	9.37	0.89	-2.15	-1.05	-0.49	0.99	sympathomimeti c
norfloxacin	5.58	8.77	-0.97	-1.8	-0.97	-1.01	-0.01	Fluoroquinolone
O,*O*-diethyl phosphate	1.95		0.45	0.26	-1.93	-1.93	-1.00	pesticide metabolite
oxalate	1.36		-0.26	-0.77	-6.88	-7.17	-2	organic acid
oxipurinol	6.25	2.09	-1.67	-2.20	-2.78	-3.21	0.05	drug metabolite
oxytetracycline	7.25	5.80	-4.54	-5.29	-4.92	-5.46	-0.71	tetracycline antibiotic
paracetamol	9.46		0.91	0.91	0.9	0.89	-0.01	aniline analgesics
paraquat			-6.70	-6.70	-6.70	-6.70	2.00	herbicide
paromomycin	12.15	9.59	-8.31	-23.47	-13.96	-11.68	4.41	aminoglycoside a ntibiotic
pefloxacin	5.55	7.01	0.26	-1.67	-0.11	-0.58	-0.68	quinolone
penicillin V	3.39		0.76	0.75	-2.64	-2.73	-1.00	penicillin
perchlorate	-7.06		-0.10	-2.47	-2.47	-2.47	-1.00	inorganic anion

analyte	strongest		log P	log D (pH)			charge pH 7.4	function
	acidic pKa	basic pK_a		1.7	7.4	8.0		
phosphate	1.8		-1.02	-1.27	-3.97	-4.46	-1.85	inorganic anion
pimaricin	3.58	9.11	-1.70	-2.44	-1.7	-1.72	-0.02	antifungal medication
piperacillin	3.49		-0.26	-0.27	-3.64	-3.75	-1.00	β-lactam antibiotic
propionic acid	4.75		0.48	0.48	-2.12	-2.59	-1.00	organic acid
quadrol	14.69	9.10	-0.89	-7.43	-2.59	-2.02	0.98	intermediate compound
ranitidine		7.80	0.99	-2.53	0.45	0.78	0.71	H_2 receptor antagonist
rantinidine *N*-oxide	14.97	3.76	-0.13	-0.24	-0.13	-0.13	0	drug metabolite
rantinidine *S*-oxide		7.69	-0.82	-4.34	-1.28	-0.99	0.66	drug metabolite
ribose	12.34		-2.94	-2.94	-2.94	-2.94	0	carbohydrates
ristocetin A	3.16	9.65	-9.20	-12.56	-9.16	-9.2	0.17	glycopeptide antibiotic
saccharine	1.94		0.45	0.28	-0.49	-0.49	-1.00	artificial sweetener
salbutamol	10.12	9.40	0.34	-2.36	-1.32	-0.77	0.99	antiasthmatic agent
sarafloxacin	5.55	8.76	0.52	-0.31	0.51	0.47	-0.01	quinolone
sorbitol	12.59		-3.73	-3.73	-3.73	-3.73	0	nutritive sweetener
sotalol	10.07	9.43	-0.40	-3.19	-2.12	-1.56	0.99	beta blocker
succinate	3.55		-0.40	-0.4	-5.47	-6.17	-1.99	organic acid
sucralose	11.91		-0.47	-0.47	-0.47	-0.47	0	artificial sweetener
sucrose	11.84		-4.53	-4.53	-4.53	-4.53	0	carbohydrates
sulfachlor-pyridazine	6.60	2.02	0.85	0.37	0.22	0.22	-0.86	sulfonamide antibiotic
sulfadiazine	6.99	2.01	0.39	-0.09	-0.05	-0.33	-0.72	sulfonamide antibiotic
sulfadimidine	6.99	2.00	0.65	0.18	0.21	-0.06	-0.72	sulfonamide antibacterial
sulfameter	7.06	1.98	0.23	-0.23	-0.18	-0.45	-0.69	sulfonamide antibacterial

analyte	strongest		log P	log D (pH)			charge pH 7.4	function
	acidic pKa	basic pK_a		1.7	7.4	8.0		
sulfamethazine	6.99	2.00	0.65	0.18	0.21	-0.06	-0.72	sulfonamide antibacterial
sulfametho-xazole	6.16	1.97	0.79	0.33	0	-0.11	-0.95	sulfonamide antibiotic
sulfate	-3.03		-0.84	-3.43	-5.59	-5.59	-2.00	inorganic anion
sulfathiazole	6.93	2.04	0.98	0.47	0.5	0.24	-0.75	sulfonamide antibiotic
tartrate	2.72		-1.83	-1.87	-7.89	-8.39	-2.00	organic acid
terbutaline	8.86	9.76	0.44	-1.89	-0.73	-0.19	0.96	beta adrenergic receptor agonists
tetracycline	7.20	6.22	-3.49	-4.22	-3.81	-4.28	-0.57	tetracycline antibiotic
tetraethyl-ammonium			-2.54	-2.54	-2.54	-2.54	1.00	other
tetramethyl-ammonium			-3.97	-3.97	-3.97	-3.97	1.00	other
tetrapropyl-ammonium			-0.45	-0.45	-0.45	-0.45	1.00	other
TFNA[8] (flonicamid metabolite)	2.62	3.99	0.80	0.44	-2.04	-2.18	-1.00	pesticide metabolite
TFNG[9] (flonicamid metabolite)	2.84	3.53	-0.50	-0.74	-3.28	-3.33	-1.00	pesticide metabolite
theophylline	7.82	-0.78	-0.77	-0.77	-0.89	-1.11	-0.28	methylx-anthine
thiamphenicol	8.75		-0.22	-0.22	-0.24	-0.28	-0.04	broadband antibiotic
thifensulfuron-methyl	5.20	1.30	0.45	-0.03	-2.03	-2.6	-1.00	herbicide
thiocyanate	0.50		0.51	-0.39	-0.64	-0.64	-1.00	other
thiosemicarbazide	14.51	3.86	-0.77	-2.89	-0.77	-0.77	0	intermediate compound
thiosulfate	-2.28		-0.10	-2.46	-2.47	-2.47	-1.00	intermediate compound
thiourea	15.22		-0.47	-0.47	-0.47	-0.47	0	intermediate compound
threonate	3.4		-2.15	-2.16	-5.55	-5.64	-1.00	sugar

analyte	strongest		log P	log D (pH)			charge pH 7.4	function
	acidic pKa	basic pK_a		1.7	7.4	8.0		
tiotropium	10.35		-1.76	-1.76	-1.75	-1.72	1.00	anticholinergics
tobramycin	12.53	9.54	-6.48	-21.64	-13.16	-10.7	4.42	aminoglycoside antibiotic
toyocamycin	12.46	6.51	-1.28	-3.26	-1.33	-1.29	0.56	drug metabolite
triethanol amine	15.12	8.44	-1.88	-5.38	-2.96	-2.45	0.92	intermediate compound
trifluoroacetic acid	0.95		0.91	0.09	-2.62	-2.62	-1.00	disinfection byproduct
triisopropanol amine	14.81	9.28	-0.63	-4.13	-2.5	-1.93	0.99	intermediate compound
trimethyl amine		9.57	0.19	-3.31	-1.97	-1.39	0.99	intermediate compound
trimethyl-sulfonium			-0.08	-0.08	-0.08	-0.08	1.00	ionic liquid
uracil 1-β-d-arabinofura-noside	9.09		-2.80	-2.80	-2.80	-2.80	0	human metabolites
vancomycin	3.38	9.90	-4.39	-7.36	-4.85	-4.39	0.89	glycopeptide antibiotic
vanillactic acid	3.37		0.72	0.71	-2.69	-2.78	-1.00	drug metabolite
vanilmandelic acid	3.11		0.44	0.42	-3.03	-3.08	-1.00	drug metabolite

[1] AMP – adenosine monophosphate; [2] AMPA – α-amino-3-hydroxy-5-methyl-4-isoxazolepropionic acid; [3] cAMP – cyclic adenosine monophosphate; [4] cCMP – cyclic cytidine monophosphate; [5] cGMP – cyclic guanosine monophosphate; [6] CMP – cytidine monophosphate; [7] GMP – guanosine monophosphate; [8] TFNA – 4-trifluoromethyl nicotinic acid; [9] TFNG – *N*-(4-trifluoromethylnicotinoyl)glycine)
